# Whole-Genome Sequencing of *Erwinia amylovora* Strains from Mexico Detects Single Nucleotide Polymorphisms in *rpsL* Conferring Streptomycin Resistance and in the *avrRpt2* Effector Altering Host Interactions

**DOI:** 10.1128/genomeA.01229-13

**Published:** 2014-01-23

**Authors:** Theo H. M. Smits, Víctor M. Guerrero-Prieto, Germán Hernández-Escarcega, Jochem Blom, Alexander Goesmann, Fabio Rezzonico, Brion Duffy, Virginia O. Stockwell

**Affiliations:** a Research Group for Environmental Genomics and Systems Biology, Institute for Natural Resources Sciences, Zurich University of Applied Sciences ZHAW, Wädenswil, Switzerland; b Plant Protection Division, Agroscope Changins-Wädenswil ACW, Wädenswil, Switzerland; c FACIATEC, University of Chihuahua, Cuauhtémoc, Chihuahua, Mexico; d Bioinformatics and Systems Biology, Justus-Liebig-Universität, Giessen, Germany; e Department of Botany and Plant Pathology, Oregon State University, Corvallis, Oregon, USA

## Abstract

We report draft genome sequences of three Mexican *Erwinia amylovora* strains. A novel plasmid, pEA78, was identified. Comparative genomics revealed an *rpsL* chromosomal mutation conferring high-level streptomycin resistance in two strains. In the effector gene *avrRpt2*, a single nucleotide polymorphism was detected that overcomes fire blight disease resistance in *Malus* × *robusta* 5.

## GENOME ANNOUNCEMENT

Fire blight caused by the enterobacterial phytopathogen *Erwinia amylovora* was reported in the major apple-growing region of Chihuahua, Mexico, in 1974 (1). Streptomycin sprays during bloom, used for decades for disease control in the United States, have proven ineffective in Mexico, where oxytetracycline and gentamicin are instead widely used (2–5). Genome sequences of three *E. amylovora* strains from Mexico were determined, providing the first genetic insights into antibiotic resistance and diversity in Mexican pathogen populations.

*E. amylovora* LA635 and LA636 were isolated from two Golden Delicious apple orchards in Cuauhtémoc County; LA637 was isolated near Creel, Guerrero County. Whole-genome sequencing (Illumina HiSeq 2x 100-bp shotgun sequencing) yielded 15,871,242 (LA635), 15,755,564 (LA636), and 16,055,090 (LA637) reads representing ~400× genome coverage. Genomes were assembled by combining *de novo* assembly using the Velvet short-read assembler plugin of the Geneious Server (Biomatters, Ltd., Auckland, NZ) and mapping against *E. amylovora* CFBP 1430 using Lasergene NGen 11 (DNASTAR, Madison, WI) with 8,000,000 reads. For each strain, a 3.8-Mb draft chromosome (8 contigs) and a circular plasmid (pEA29) were assembled. All sequences were annotated automatically using GenDB (6) with manual optimization (7). Plasmid pEA29 from Mexican strains shares >99% sequence identity (100% coverage) with pEA29 in other *E. amylovora* genotypes (8). We identified a novel 78-kb plasmid (pEA78) in LA637.

Comparative analysis using EDGAR (9) confirmed the highly conserved genome of this pathogen (7) but revealed single nucleotide polymorphisms (SNPs) with phenotypic effects. In LA635 and LA637, an SNP detected in the *rpsL* codon 43 substitutes arginine for lysine in ribosomal protein S12 and confers high-level resistance to streptomycin (>1,000 µg ml^–1^) (10), the most widely used antibiotic in plant agriculture. This chromosomal mutation is the predominant *E. amylovora* resistance mechanism, typically appearing after years of intensive application with long persistence in populations (11). Resistance detection despite infrequent streptomycin use in Mexico (2) explains the observed inefficacy of streptomycin (3) but underscores a need for proactive pesticide use strategies to avoid similar resistance evolution against oxytetracycline and gentamicin, which are currently relied upon.

An SNP in the type III secretion system effector *avrRpt2*, resulting in the substitution of cysteine with serine at position 156, was detected in each strain. The identical variant *avrRpt2* occurs in strains from Ontario, Michigan, and West Virginia but not elsewhere. This mutation enables *E. amylovora* to overcome the unique disease resistance recently described in *Malus* × *robusta* 5, a wild apple used in breeding programs (12). Genotyping based on clusters of regularly interspaced short palindromic repeat (CRISPR) sequences (13) and the *avrRpt2* SNP (12) suggest northeastern North America as the origin of the fire blight pathogen in Mexico (2, 3).

### Nucleotide sequence accession numbers.

Draft chromosome sequences of *E. amylovora* strains LA635, LA636, and LA637 were deposited at EMBL-EBI under accession numbers CBVS010000001 through CBVS010000008, CBVT010000001 through CBVT010000008, and CBVU010000001 through CBVU010000008, respectively. The finished plasmid sequences received accession numbers HG793096 (LA635 pEA29), HG793097 (LA636 pEA29), HG793098 (LA637 pEA29), and HG793099 (LA637 pEA78).
